# Retraction Note: HDAC6 inhibitor TST strengthens the antiproliferative effects of PI3K/mTOR inhibitor BEZ235 in breast cancer cells via suppressing RTK activation

**DOI:** 10.1038/s41419-019-1866-9

**Published:** 2019-08-22

**Authors:** Shixiu Sun, Yujie Zhang, Jianchao Zheng, Biao Duan, Jie Cui, Yan Chen, Wenjie Deng, Bixing Ye, Lei Liu, Yongchang Chen, Jun Du, Luo Gu

**Affiliations:** 1 Department of Physiology, Nanjing Medical University, Nanjing, Jiangsu 211166 China; 2 Key Laboratory of Cardiovascular & Cerebrovascular Medicine, School of Pharmacy, Nanjing Medical University, Nanjing, Jiangsu 211166 China; 3 Jiangsu Key Lab of Cancer Biomarkers, Prevention and Treatment, Collaborative Innovation Center For Cancer Personalized Medicine, Nanjing Medical University, Nanjing, Jiangsu 211166 China; 4 BGI Education Center, University of Chinese Academy of Sciences, Shenzhen, Guangdong 518083 China; 5 Department of Physiology, Xuzhou Medical University, Xuzhou, Jiangsu 221004 China; 6 Department of Biochemistry and Molecular Biology, Nanjing Medical University, Nanjing, Jiangsu 211166 China; 7 Department of Gastroenterology, The First Affiliated Hospital of Nanjing Medical University, Nanjing, Jiangsu 210029 China; 8 Department of Physiology, School of Medicine, Jiangsu University, Zhenjiang, Jiangsu 212013 China

**Retraction Note to: Cell Death & Disease** 9:929

10.1038/s41419-018-0931-0 published online 11 September 2018

This article was published in error and has been retracted at the request of the authors. During the proofing stage, the authors found that some lanes in Figs. 2b, 2h, 3h and S4a were not the appropriate ones. Shixiu Sun, Yujie Zhang, Jianchao Zheng, Biao Duan, Yan Chen, Wenjie Deng, Bixing Ye, Lei Lui, Yongchang Chen, Jun Du and Luo Gu agree with this retraction. We have been unable to contact Jie Cui. The authors have been given the opportunity to submit a revised manuscript for peer review.

